# The efficacy of antidepressant medication versus interpersonal psychotherapy for depression in middle-aged women: a systematic review and individual participant data meta-analysis

**DOI:** 10.1007/s00737-026-01730-1

**Published:** 2026-07-20

**Authors:** Sanne N.E. van Haaren, Zachary D. Cohen, Jasmijn Breunese, Frederik J. Wienicke, John C. Markowitz, Erica S. Weitz, Steven D. Hollon, Dillon T. Browne, Paula Rucci, Carolina Corda, Marco Menchetti, Myrna M. Weissman, R. Michael Bagby, Lena C. Quilty, Marc B.J. Blom, Mario Altamura, Ingo Zobel, Elisabeth Schramm, Carlos Gois, Jos W.R. Twisk, Pim Cuijpers, Ellen Driessen

**Affiliations:** 1 Pro Persona Mental Health Care, Wolfheze, Netherlands; 2 Department of Psychology, University of Arizona, Tucson, USA; 3 Behavioural Science Institute, Department of Clinical Psychology, Radboud University, Nijmegen, Netherlands; 4 Vagelos College of Physicians & Surgeons, Columbia University, New York, USA; 5 New York State Psychiatric Institute, New York, USA; 6 Department of Psychiatry, University of Pennsylvania, Philadelphia, USA; 7 Department of Psychology, Vanderbilt University, Nashville, USA; 8 Department of Psychology, University of Waterloo, Waterloo, Canada; 9 Department of Biomedical and Neuromotor Sciences, University of Bologna, Bologna, Italy; 10 Department of Experimental, Diagnostic and Speciality Medicine, University of Bologna, Bologna, Italy; 11 Department of Psychology and Psychiatry, University of Toronto, Toronto, Canada; 12 Campbell Family Mental Health Research Institute, Centre for Addiction and Mental Health, Toronto, Canada; 13 Department of Psychiatry, University of Toronto, Toronto, Canada; 14 Parnassia Groep, The Hague, Netherlands; 15 Department of Clinical and Experimental Medicine, University of Foggia, Foggia, Italy; 16 Psychology School, Hochschule Fresenius, University of Applied Sciences Berlin, Berlin, Germany; 17 Faculty of Medicine, Medical Centre, Department of Psychiatry and Psychotherapy, University of Freiburg, Freiburg, Germany; 18 Department of Psychiatry, University of Lisbon, Lisbon, Portugal; 19 Department of Epidemiology and Data Science, Amsterdam UMC Location VUmc, Amsterdam, Netherlands; 20 Department of Clinical, Neuro and Developmental Psychology, Amsterdam Public Health Institute, Vrije Universiteit Amsterdam, Amsterdam, Netherlands

**Keywords:** Middle-aged women, Depression, Antidepressant medication, Interpersonal psychotherapy

## Abstract

**Purpose:**

Depression is highly prevalent in middle-aged women (MAW), but head-to-head comparisons of first-line interventions in this population are scarce. This systematic review and individual participant data (IPD) meta-analysis examined the comparative efficacy of antidepressant medication (ADM) and interpersonal psychotherapy (IPT) for MAW (aged 40–64) versus other adults (men and non-MAW) on depression, quality of life, and interpersonal problem measures.

**Methods:**

PubMed, PsycINFO, Embase, and the Cochrane Library were searched January 1, 2025 to identify randomized trials comparing ADM and IPT for adults with depression. IPD were requested and analyzed using mixed-effects models.

**Results:**

IPD were obtained from 9 of the 15 (60%) identified studies, comprising 1,490 participants (527 MAW, 963 other adults). MAW status did not significantly moderate ADM versus IPT comparative efficacy on depression (*p* = .10) and interpersonal problems (*p* = .29) outcomes. The two treatments did not significantly differ in treatment effect in the MAW group (depression: *b* = -0.03, *p* = .76; interpersonal problems: *b* = -0.20, *p* = .31). MAW status significantly moderated comparative efficacy on quality of life measures (*b *= 0.37, *p* = .02). Whereas IPT was non-significantly more efficacious than ADM for MAW (*b* = -0.18, *p* = .15), ADM were non-significantly more efficacious than IPT for other adults (*b* = 0.19, *p* = .051).

**Conclusion:**

This is the first IPD meta-analysis directly comparing depression treatments in MAW. The absence of treatment effect differences indicates that other factors (e.g., patient preference) may guide choice between IPT and ADM in this population.

**Supplementary Information:**

The online version contains supplementary material available at https://doi.org/10.1007/s00737-026-01730-1.

## Introduction

Depression affects more than 332 million people worldwide, ranking it among the most prevalent mental disorders. Imposing significant costs for individuals, their families, and society at large, depression is considered the leading cause of disability worldwide (James et al. 2018, World Health Organization 2025). On average, women are 1.5 times as likely as men (World Health Organization 2025) to experience depression and middle-aged women (MAW) are more likely to have depressive episodes than women in any other age group (Angst et al. 2002; Arias de La Torre et al. 2021; Freeman et al. 2004; Kessler et al. 1994; Kulkarni et al. 2024; Llaneza et al. 2012; Soares and Shea 2021).

Middle age, defined as ages 40–64 (Thomas et al. 2018), is a distinct life stage characterized by biological, social, and psychological transitions, which can co-occur and are associated with the onset of depressive symptoms and depression in women (Dennerstein and Soares 2008). Risk factors for depression in MAW can be either continuous or periodic (Soares and Shea 2021). Continuous risk factors (i.e., not specific to middle age) include health, demographic, socioeconomic, and psychosocial factors. In contrast, periodic risk factors relate to specific times or contexts and for MAW include peri-menopausal phenomena (e.g., hormonal fluctuations, sleeping problems, and vasomotor symptoms), stressful life events (e.g., role change, grief, loss, separation), and multiple simultaneous roles (concurrent management of occupational, familial, and social responsibilities (Dare et al. 2011; Thomas et al. 2018). The combination of continuous and periodic risk factors create a multifactorial etiology for depression in MAW, making it difficult to treat (Bromberger et al. 2004).

Antidepressant medications (ADM) and psychological interventions such as interpersonal psychotherapy (IPT) are empirically supported first-line interventions for mild to moderate depression (American Psychological Association 2019; National Institute for Health and Care Excellence 2022; Canadian Network for Mood and Anxiety Treatments 2023). ADM and IPT are presumed to align well with the specific periodic risk factors during this stage of life. ADM positively affect sleep, vasomotor symptoms, and mood in women undergoing menopause (Maki et al. 2019; Khushboo and Sharma 2017; Freeman et al. 2006). Additionally, ADM may be effective in reducing depressive symptoms resulting from an increased sensitivity to hormone changes to which some, but not all, women are vulnerable during the various menopausal stages (Schmidt et al. 1998; Bloch et al. 2000; Lokuge et al. 2011). IPT is a structured, time-limited psychotherapy specifically developed to treat major depression, focusing on the connection of mood and stressful life events such as loss, separation, interpersonal disputes, and other significant life changes. IPT has been shown to help individuals build social skills and mobilize social supports (Weissman et al. 2018). Although not specifically designed for middle age, its focus on interpersonal transitions and role changes are highly relevant to female depression in this age group.

An increasing number of studies have examined the treatment of depressive symptoms occurring during the perimenopausal transition, and recommend ADM as an effective therapeutic option for women in this phase (Freeman et al. 2017; Frey et al. 2013; Gambacciani et al. 2003; Kornstein et al. 2010; Maki et al. 2019; Soares et al. 2009, 2010; Soares 2023; Zhou et al. 2021), with no evidence supporting one ADM over others (Soares 2023). First-line psychotherapeutic interventions for depression, such as IPT, have not been specifically studied in women with hormone-driven depression or in MAW, but are recommended based on their established positive effects in the general population (Maki et al. 2019; Soares 2023). To the best of our knowledge, no studies have directly compared the efficacy of ADM and IPT in middle-aged depressed women.

The World Health Organization (WHO) highlights the need for further research into gender-specific antidepressant treatment approaches (WHO 2022). Given the high prevalence of depression among MAW and the treatment challenges posed by its multifactorial etiology, this study aimed to examine the comparative efficacy of ADM and IPT for MAW versus other adults (men and non-MAW). In line with recent calls to evaluate multiple domains of functioning, we operationalized efficacy as changes in depressive symptoms, quality of life, and interpersonal problems (Cuijpers 2020; Hirschfeld et al. 2000; Kennedy et al. 2001), recognizing that periodic risk factors in MAW may also substantially affect psychosocial functioning. As such, the present study addressed the following research questions formulated in PICOT format: how does IPT (I) compare to ADM (C) in terms of depressive symptom severity, quality of life, and interpersonal problems (O) after acute-phase treatment (T) in MAW versus other adults with depression (P)?

To address questions of efficacy of treatments for depression in women in mid-life with robust methods, we conducted an individual participant data (IPD) meta-analysis. In contrast to conventional meta-analysis, which is based on study-level information extracted from publications, IPD meta-analysis combines participant-level datasets of the included studies, offering several advantages. Data analysis methods can be standardized across studies, results of primary studies can be verified, and data not reported in original publications can be analyzed, yielding more precise effect estimates (Stewart and Parmar 1993; Riley et al. 2010). Most importantly, IPD meta-analyses allows examination of potential moderators on the participant-level with increased statistical power compared to both single randomized clinical trials (RCTs) and conventional meta-analyses due to larger sample sizes (Tudur Smith et al. 2016). This design enables differentiating between MAW and other adults at the individual participant level, allowing us to estimate and compare treatment effects in these groups. Because of these advantages, we considered IPD meta-analysis the optimal method to examine our research questions.

## Methods

### Design

This IPD meta-analysis is part of a larger project with a published detailed protocol (Driessen et al. 2021), listed on the PROSPERO International prospective register of systematic reviews (No. CRD42020219891). The larger project's outcomes have been described elsewhere (Cohen et al. 2024). We pre-registered the current study on Open Science Framework (https://osf.io/m425h).

### Eligibility criteria

We included RCTs comparing ADM and IPT in the acute-phase treatment of adults with depression. We defined ADM as any oral antidepressant medication within the therapeutic dose range: e.g., selective serotonin reuptake inhibitors, tricyclic antidepressants, and monoamine oxidase inhibitors. An intervention was considered IPT if it was based on the manuals developed by Klerman and Weissman (Klerman et al. 1984; Weissman et al. 2018) or on the manual for the shortened version, interpersonal counseling (IPC; Weissman et al. 2014). Sessions could be delivered in any setting, format, or time frame so long as a clinician provided therapy. Participants had to be 18 years or older and were considered to have a depression if they either met specified diagnostic criteria (e.g., Diagnostic and Statistical Manual of Mental Disorders [DSM]) for a unipolar mood disorder, or reported an elevated score above the ‘no depression’ cut-off on a standardized measure of depressive symptoms. Somatic and comorbid psychiatric disorders were allowed (Driessen et al. 2021).

### Study selection

Studies were identified by searching the METAPSY database of psychotherapy RCTs for depression, which derives from comprehensive literature searches in the bibliographic databases PubMed, PsycINFO, Embase, and the Cochrane Library (exact terms are available; https://osf.io/nv3ea/). Searches were conducted up to January 1st, 2025. Two independent raters assessed study eligibility in each phase with disagreement being resolved in consensus (Driessen et al. 2021).

### Data collection

Authors of the included studies were contacted using a multi-step protocol and invited to contribute the anonymized participant-level data of their studies. The request encompassed all demographic, clinical, and psychological participant characteristics assessed before treatment start as well as all outcome measures assessed on all time points included in the study. We conducted data integrity checks to assess whether the data set received matched the data reported in the publication, included all outcome variables, and contained no invalid items on the variables of interest for the current study (Driessen et al. 2021).

**Table 1:** Characteristics of included studies

Study	*N*(ADM/IPT)^a^	Country	Depression diagnosis	ADM type	IPT Format	*N* sessions
Altamura et al., 2017	55 (28/27)	Italy	Major Depressive Disorder (DSM- 5); HAMD ≥ 8	SSRI	Individual^b^	6
Blom et al., 2007	97 (47/50)	Netherlands	Major Depressive Disorder (DSM- 4); HAMD ≥ 14	Other^c^	Individual	12
Browne et al., 2002	460 (229/231)	Canada	Dysthymic disorder, with or without Major Depressive Disorder (DSM-4)	SSRI	Individual	12
Elkin et al., 1989	126 (63/63)	USA	Major Depressive Disorder (RDC); HAMD ≥ 14	TCA	Individual	16–20
Finkenzeller et al., 2009	51 (24/27)	Germany	Depressive disorder (ICD-10); HADS > 7; HAMD ≥ 14	SSRI	Group	8–16
Frank et al., 2011	291 (142/149)	USA; Italy	Major depressive episode (DSM-4); HAMD ≥ 15	SSRI	Individual	12
Gois et al., 2014	34 (17/17)	Portugal	Major Depression (DSM-4); HADS ≥ 7; MADRS ≥ 17	SSRI	Individual	12
Menchetti et al., 2014	287 (144/143)	Italy	Major depressive episode (DSM-4); HAMD ≥ 13	SSRI	Individual^b^	6–8
Quilty et al., 2008	135 (72/63)	Canada	Major depressive disorder (DSM-4)	SSRI, SNRI, MAOI, Other^d^	Individual	16–20

Note. *ADM* antidepressant medication, *DSM* diagnostic and statistical manual of mental disorders, *HADS* hospital anxiety and depression scale,* HAM-D* Hamilton Depression rating scale, *ICD*-10 international statistical classification of diseases and related health problems, 10th edition, *IPD* individual participant data, *IPT* interpersonal psychotherapy, *MAOI *monoamine oxidase inhibitor*, MAW* middle-aged women,* MADRS* montgomery åsberg depression rating scale, *N* number of participants, Nsessions number of IPT or IPC sessions, *RDC* research diagnostic criteria, *SNRI* selective serotonin and noradrenalin reuptake inhibitor,* SSRI* selective serotonin reuptake inhibitor, *TCA* tricyclic antidepressant

^a^ Number of patients receiving ADM or IPT monotherapy

^b^ Therapy format included interpersonal counselling (IPC)

^c^ Nefazodone

^d^ Bupropion

Two independent raters assessed risk-of-bias in the included studies at outcome level using the Cochrane Collaboration’s risk-of-bias tool for randomized trials (version 2; Higgins et al. 2022). Disagreements were resolved by consensus or discussed with a third rater (Driessen et al. 2021).

### Measures

All instruments explicitly measuring depressive symptom level, quality of life, and interpersonal problems were included. Because different instruments were used to assess these constructs, scores for each outcome were standardized by conversion to z-scores within time-point and study (Driessen et al. 2021). MAW were defined as participants who self-reported as women in the age range 40–64 years (Thomas et al. 2018).

**Table 2:** Overview of outcome measures used by the included studies

		Included Studies								
Outcome	Measure	Altamura et al., 2017	Blom et al., 2007	Browne et al., 2002	Elkin et al., 1989	Finkenzeller et al.,^2009^	Frank et al., 2011	Gois et al., 2014	Menchetti et al., 2014	Quilty et al., 2008
Depression	HAM-D-17		x		x	x	x			x
	HAM-D-21	x							x	
	MADRS			x				x		
Quality of Life	WHOQoL-BREF								x	
	Q-LES-Q						x			
Interpersonal problems	IIP-32									x
	IPS		x							

Note. *HAM-D* Hamilton Depression rating scale, 17 and 21 items; IIP-32 32-item Inventory of Interpersonal Problems; IPS Interpersonal Sensitivity, *MADRS* Montgomery Åsberg Depression Rating Scale, *Q-LES*-Q Quality of Life Enjoyment and Satisfaction Questionnaire, WHOQoL-BREF World Health Organization Quality of Life Scale

### Data analysis

One-stage IPD meta-analyses using mixed-effects models with a three-level structure (study, participant, repeated measures) and restricted maximum likelihood were conducted in the R (version 4.0.3; R Core Team, 2020) lme4 package (version 1.1–27.1). Following each analysis, we visually inspected the plotted (standardized) residuals to ensure that the assumption of normality was not violated.

Following previous IPD meta-analyses (Cohen et al. 2024; Driessen et al. 2021), our data-analysis strategy followed the approach recommended by Twisk et al. (2018, Eq. 2c) to account for baseline differences between treatment conditions in the outcome variable. We estimated separate models for the three outcome measures. Each model included main effects for time and MAW, a time-by-treatment interaction, a time-by-MAW interaction, and a time-by-MAW-by-treatment 3-way interaction. Random intercepts for study and participants were estimated to account for clustering of, respectively, participants in studies and measurements in participants. In these models, the 3-way interaction indicates a moderator effect, i.e., a difference in the comparative efficacy of ADM versus IPT between MAW and other adults. To correct for multiple testing, a Bonferroni correction was applied. *P* values < 0.017 (3 tests) were thereby considered to indicate a significant moderator effect. The time-by-treatment interaction indicates the comparative treatment effect of ADM and IPT within the relevant subgroup (i.e., MAW or other adults). 

The mixed-effects models used all available outcome observations across time points, that is, baseline and post-treatment. Participants with an available outcome value at either baseline or post-treatment were therefore included in the models, even if they had a missing value at the other time point, provided that MAW status could be determined. Participants with no available outcome measurement at any time point were excluded.

To explore the potential effects of the different menopausal stages, we conducted pre-specified sensitivity analyses, repeating analyses for the outcome depression for the MAW age ranges; 40–48 year (peri-menopause), 49–53 year (menopause) and 54–64 year (post-menopause) (Palacios et al. 2010; Thomas et al. 2001; Stolk et al. 2012; Vivian-Taylor and Hickey 2014). We also conducted two additional post-hoc sensitivity analyses: one repeating the main analyses restricted to studies in which the ADM condition consisted of SSRIs, and one restricted to studies including unselected adult samples with fully syndromal major depressive disorder.

## Results

### Included Studies

**Fig. 1:**
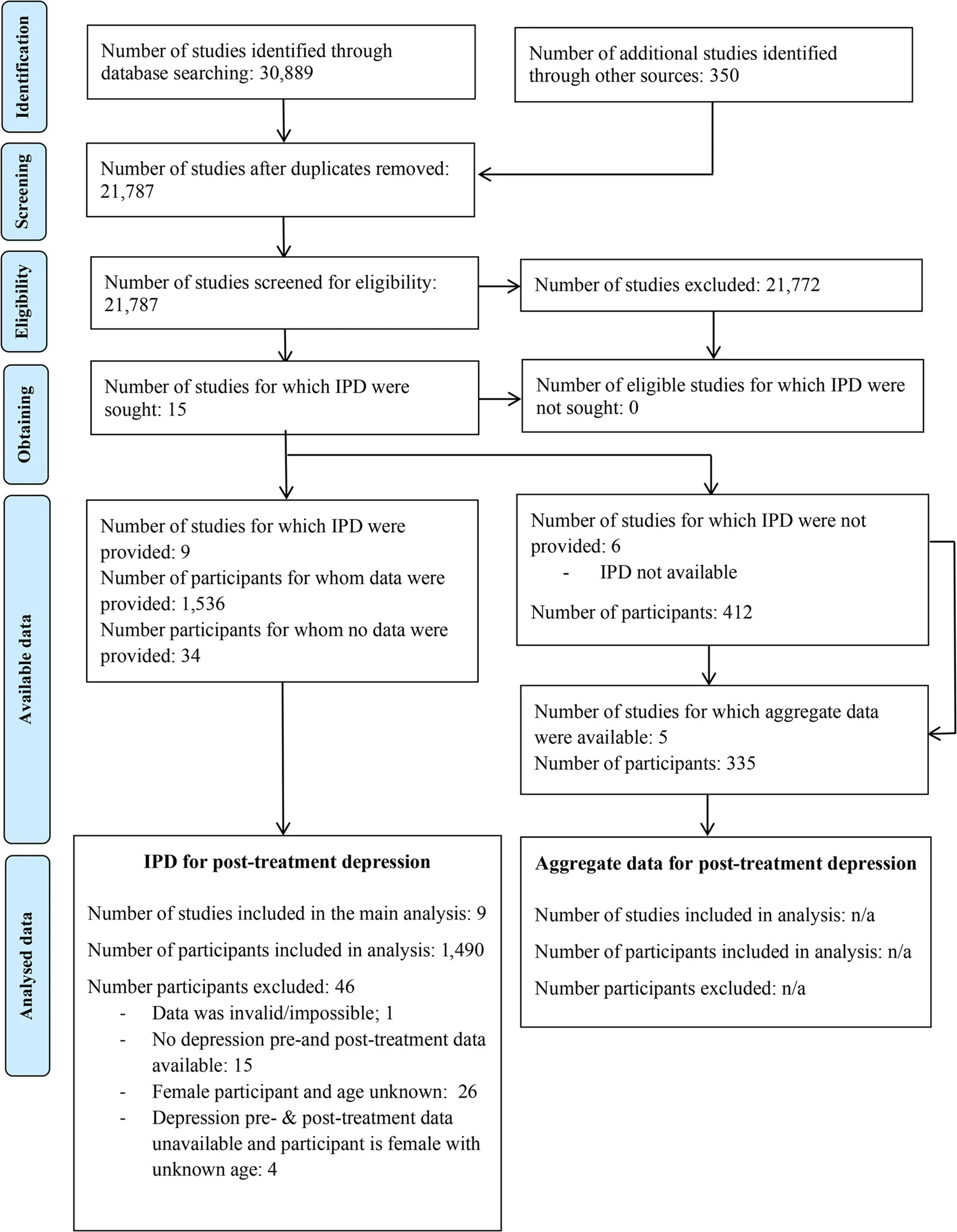
The PRISMA Individual Participant Data flow diagram

Figure 1 illustrates the PRISMA flow diagram. The systematic literature search identified 15 eligible studies (*n *= 1,948). IPD were obtained for 9 studies, including 1,536 (78.9%) participants (ADM: *n* = 766, 49.9%; IPT: *n* = 770, 50.1%).

Table 1 details study characteristics and an overview of study outcomes is provided in Table 2. Inclusion criteria for depression primarily involved a DSM diagnosis of major depressive disorder and a Hamilton Depression Rating Scale (HAM-D) score indicating the likelihood of clinically significant depressive symptoms. One study included individuals with dysthymic disorder. Seven studies addressed unselected adult populations, whereas two focused on specific groups: post-stroke depression and type 2 diabetes mellitus. Seven studies examined IPT (8–20 sessions) and two IPC (6–8 sessions), with all but one using an individual treatment format. ADM were mainly selective serotonin reuptake inhibitors.

In studies collecting these data, participants’ mean baseline HAM-D score was 18.9 (*SD* = 4.43, *N* = 999) and mean duration of the current depressive episode was 42.62 (*SD* = 54.13, *N* = 569) weeks. Based on observed data, response rates, being defined as ⩾50% symptom reduction from pre- to post-treatment on the primary continuous depression measure according to the component study authors, were 49.5% (*N* = 310/626) in the IPT and 55.2% (*N* = 341/618) in the ADM conditions. All-cause dropout, defined as having no available post-treatment depression score, occurred in 268 of 1,536 participants (17.4%) and was similar across conditions: 127 of 766 in ADM (16.6%) and 141 of 770 in IPT (18.3%). Studies did not consistently collect information on cause-specific (e.g., treatment) dropout.

Forty-six participants were excluded from the analyses because of missing pre-and post-treatment depression scores and/or age values (*n* = 45) and invalid depression scores (*n* = 1). The current study therefore included 1,490 participants: 527 MAW (40–48 years: *n =* 236; 49–53 years: *n =* 137, and 54–64 years: *n =* 154) and 963 other adults (452 men and 511 women outside the middle-age range).

### Bias assessments

Outcomes of the risk-of-bias assessments appear in Table 3. Overall risk-of-bias was of “some concern” for the outcome domain depression and “high” for quality of life and interpersonal problems. The three main sources of risk-of-bias were lack of an available prespecified research plan (selection of the reported results), reliance on outcomes assessed by self-report instruments (bias in outcome measurement), and failure to report results for the full intention-to-treat sample (missing outcome data).

**Table 3:** Risk-of-bias per outcome domain of included studies for which IPD were obtained

Outcome domain	Study	Randomization process	Deviations from the intended intervention	Missing outcome data	Measurement of the outcome	Selection of the reported results	Overall
Depression	Altamura et al., 2017 Blom et al., 2007 Browne et al., 2002 Elkin et al., 1989 Finkenzeller et al., 2009 Frank et al., 2011 Gois et al., 2014 Menchetti et al., 2014 Quilty et al., 2008	+ + + + + + + + +/-	+ + + +/- + + + +/- +	+ - + + + - + + +	+ + + + + + + + -	+/- +/- +/- +/- +/- +/- +/- + +/-	+/- +/- +/- +/- +/- +/- +/- +/- -
Quality of Life	Frank et al., 2011 Menchetti et al., 2014	+ +	+ +/-	- +	- -	+/- +/-	- -
Interpersonal problems	Blom et al., 2007 Quilty et al., 2008	+ +/-	+ +	- -	- -	+/- +/-	- -

Note. + = low risk of bias; +/- = some concerns; - = high

### IPD meta-analyses

Results of the moderator models are presented in Table 4. MAW status did not moderate the comparative efficacy of ADM and IPT on post-treatment measures of depressive symptoms (*b* = 0.19, 95% CI [-0.03, 0.40], *p* = .10). No significant difference in treatment efficacy was found for MAW (*b* = -0.03, 95% CI [-0.20, 0.15], *p* = .757). This result was replicated in all sensitivity analyses (Table 5). However, ADM was more efficacious than IPT for other adults (*b* = 0.16, 95% CI [0.02, 0.29], *p* = .020).

**Table 4:** MAW-status as a moderator of ADM versus IPT comparative efficacy

Outcome		k	*N*	b	95% CI	*p*
**Depression**		9	1,490			
MAW				-0.03	-0.20, 0.15	0.757
Other Adults				0.16	0.02, 0.29	0.020
**Quality of life**ª		2	567			
MAW				**-0.18**	**-0.41**,** 0.07**	**0.149**
Other Adults				**0.19**	**-0.001**,** 0.37**	**0.051**
**Interpersonal problems**		2	187			
MAW				-0.20	-0.58, 0.18	0.306
Other Adults				0.05	-0.22, 0.33	0.701

Note. *ADM* antidepressants medication, *IPT* interpersonal psychotherapy, *MAW* middle-aged women

Positive signs indicate better outcomes in the ADM than in the IPT condition and negative signs indicate better outcomes in the IPT than in the ADM condition. Statistical significance (*p*<.05) of the time-by-moderator-by-treatment 3-way interaction is marked by bold printed numbers, indicating a differential treatment efficacy between the MAW and Other Adults groups

^a^ b = 0.37, 95% CI [0.05, 0.68], *p* = .019

**Table 5:** MAW-status as moderator of ADM versus IPT comparative efficacy on depression outcomes – sensitivity analyses

Moderator	k	*N*	b	95% CI	*p*
**Perimenopausal**	9	1,490			
Women 40–48 years old			-0.14	-0.40, 0.12	.308
Other Adults*			0.14	0.02, 0.25	.022
**Menopausal**	9	1,490			
Women 49–53 years old			0.05	-0.30, 0.40	.781
Other Adults*			0.09	-0.02, 0.20	.100
**Postmenopausal**	9	1,490			
Women 54–64 years old			0.07	-0.25, 0.41	.650
Other Adults*			0.09	-0.02, 0.21	.112
**SSRI-only studies**	6	1,142			
MAW			-0.03	-0.21, 0.17	.776
Other Adults			0.14	-0.01, 0.29	.073
**MDD general adult sample**	6	966			
MAW			-0.13	-0.35, 0.10	.267
Other Adults			0.11	-0.05, 0.27	.179

Note. *ADM* antidepressant medication, *IPT* interpersonal psychotherapy, *MAW* middle-aged women, *MDD *major depressive disorder

Positive signs indicate better outcomes in the ADM than in the IPT condition and negative signs indicate better outcomes in the IPT than in the ADM condition

* Men and all women outside the specified age range

Comparative ADM-IPT efficacy on post-treatment outcomes of interpersonal problems was not moderated by MAW status (*b* = 0.25, 95% CI [-0.22, 0.73], *p* = .29). No significant difference in treatment efficacy emerged either for MAW or other adults (MAW: *b* = -0.20, 95% CI [-0.58, 0.18], *p* = .306; Other adults: *b* = 0.05, 95% CI [-0.22, 0.33], *p* = .701).

MAW status did moderate the comparative efficacy of ADM and IPT on post-treatment quality of life measures (*b* = 0.37, 95% CI [0.05, 0.68], *p* = .019). While for MAW, IPT was non-significantly more efficacious than ADM (*b* = -0.18, 95% CI [-0.41, 0.07], *p* = .149), ADM was non-significantly more efficacious than IPT for other adults, with a *p*-value that just exceeded the 0.05 significance threshold (*b* = 0.19, 95% CI [-0.001, 0.37], *p* = .051).

## Discussion

This IPD meta-analysis aimed to advance knowledge on gender-specific treatment options for middle-aged depressed women by examining the comparative efficacy of ADM and IPT for women in this age group versus other adults. Although MAW-status did not moderate the ADM versus IPT comparative efficacy on depression and interpersonal problems outcomes, it significantly moderated post-treatment quality of life measures. Among MAW, no significant differences were found between ADM and IPT in post-treatment depressive symptoms, quality of life, and interpersonal problems.

While ADM are effective in the treatment of depression, including in MAW during menopause (Maki et al. 2019; Soares 2023), evidence regarding the efficacy of IPT for middle-aged depressed women remains limited, as studies specifically targeting this population are scarce. The present findings suggest that IPT may be as effective as ADM across multiple outcome domains in MAW with depression. This is in line with previous research supporting the efficacy of IPT for depression more broadly (Cuijpers et al. 2016; de Mello et al. 2005; Markowitz and Weissmann 2012). Together, these findings support IPT as a viable treatment option for MAW with depression, particularly for patients who prefer psychotherapy over pharmacological treatment or for whom ADM may be less suitable.

A moderating effect was observed indicating differences in the comparative efficacy of ADM and IPT between MAW and other adults on quality of life outcomes. Specifically, in the MAW group, no differences in treatment effect between ADM and IPT were observed whereas in the other adults group, ADM was more efficacious than IPT with a small effect size and a marginal significance level (*p* = .051). Given the small number of studies on which this analysis is based (*k* = 2), we consider this finding preliminary and in need of replication in larger samples.

### Strengths and limitations

This study possesses several strengths. First, the IPD meta-analysis design enabled us to standardize data analysis methods across studies, verify results of the primary studies, and include data not reported in publications, providing more precise effect estimates than conventional meta-analysis. Second, this design allowed examination of potential moderators on the participant-level, with a higher statistical power than in single RCTs and conventional meta-analyses due to larger sample sizes (Tudur Smith et al. 2016). As a consequence, we were able to examine treatment effects in a relatively large sample of MAW and compare these to other adults. This is the first study to examine comparative antidepressant treatment effects for MAW, thereby answering the WHO call for examining age- and gender-specific interventions.

This study has several limitations. First, the included studies were not free from risk of bias. Although IPD meta-analyses help mitigate some risk-of-bias issues by allowing more detailed and standardized data analysis, concerns remain regarding the inclusion of self-report measures, which could impact outcome measurement and reporting. Second, not all included studies assessed quality of life and interpersonal problems, with these outcomes being measured in only two studies. Although the sample size was large enough to detect a moderation effect for quality of life measures, this might not have been the case for interpersonal problems. Third, the participating studies were conducted exclusively in Western countries, which may limit the generalizability of the results to other cultural contexts. Fourth, the sensitivity analyses were based on females’ age as a proxy for menopause stage. These do not necessarily translate to participants’ true menopause stage and the age range for menopause may have been too broad. Fifth, although the included studies shared similarities in terms of target group, depression inclusion criteria, and IPT format, they also differed, for instance with respect to treatment length and antidepressant type. Although restricting the analyses to studies examining SSRIs only did not change the pattern of results, these differences may be potential sources of heterogeneity.

### Clinical and research Implications

The findings of this study suggest important clinical implications for the treatment of depression in MAW. We found no significant outcome differences between IPT and ADM on all outcome measures among MAW. This suggests that clinicians can use either ADM or IPT in this population, allowing them to tailor treatment based on patient preferences, side effects, and accessibility.

We recommend that future research systematically tracks each female exposure to both periodic and continuous risk factors for depression, including menopausal stage and life events. This approach would permit a more granular analysis of the specific conditions that contribute to depressive episodes, facilitating identification of optimal treatment methods. Inasmuch as ADM and IPT target different risk factors, research for MAW needs to determine whether combined treatment can improve outcomes. By maintaining detailed records of risk factors, we can better understand interactions between various determinants of depression, ultimately producing personalized, more effective therapeutic interventions. Finally, it is advisable to broaden future research to encompass the full range of first-line treatment interventions for depression in MAW.

This study attempted to provide more age- and gender-specific treatment recommendations for MAW with depression, as the WHO advises. Hormonal fluctuations make the female body unique and affect biological, psychological, and social factors. It is crucial that future research continues to consider these aspects.

## Supplementary Information

Below is the link to the electronic supplementary material.

Supplementary Material (r 18.9 KB)

## Data Availability

The collective de-identified individual participant database developed for this study, as well as a data dictionary and relevant related documents (e.g., study protocol) are available for use by other researchers with publication of this manuscript. Requests can be made with the corresponding author (ellen.driessen@ru.nl). Access (with limited investigator support) will be granted after approval of a study proposal by all authors and a signed data access agreement. The code for this study will be available as supplementary material.
